# Microbiome-Metabolome Responses in the Cecum and Colon of Pig to a High Resistant Starch Diet

**DOI:** 10.3389/fmicb.2016.00779

**Published:** 2016-05-26

**Authors:** Yue Sun, Yong Su, Weiyun Zhu

**Affiliations:** Jiangsu Key Laboratory of Gastrointestinal Nutrition and Animal Health, College of Animal Science and Technology, Nanjing Agricultural UniversityNanjing, China

**Keywords:** hindgut, metabolite profiles, microbiota, pig, resistant starch

## Abstract

Currently, knowledge about the impact of long-term intake of high resistant starch diet on pig hindgut microbiota and metabolite profile is limited. In this study, a combination of the pyrosequencing and the mass spectrometry (MS)-based metabolomics techniques were used to investigate the effects of a raw potato starch (RPS, high in resistant starch) diet on microbial composition and microbial metabolites in the hindgut of pig. The results showed that *Coprococcus, Ruminococcus*, and *Turicibacter* increased significantly, while *Sarcina* and *Clostridium* decreased in relative abundances in the hindgut of pigs fed RPS. The metabolimic analysis revealed that RPS significantly affected starch and sucrose metabolites, amino acid turnover or protein biosynthesis, lipid metabolites, glycolysis, the pentose phosphate pathway, inositol phosphate metabolism, and nucleotide metabolism. Furthermore, a Pearson's correlation analysis showed that *Ruminococcus* and *Coprococcus* were positively correlated with glucose-6-phosphate, maltose, arachidonic acid, 9, 12-octadecadienoic acid, oleic acid, phosphate, but negatively correlated with α-aminobutyric acid. However, the correlation of *Clostridium* and *Sarcina* with these compounds was in the opposite direction. The results suggest that RPS not only alters the composition of the gut microbial community but also modulates the metabolic pathway of microbial metabolism, which may further affect the hindgut health of the host.

## Introduction

Studies of the gut microbial ecosystem have identified that the human intestine harbors a complex and diverse community of microbiota comprising approximately 100 trillion microbes, including bacteria belonging to several phyla with more than 1000 species (Rajilić−Stojanović et al., 2007; Zoetendal et al., 2008). Many of these microbial genes are involved in the main metabolic pathways, such as carbon metabolism and amino acid synthesis (Li et al., 2008). The gut microbes and their metabolism products are important environmental factors that affect the immune system, obesity, cardiovascular disease, and brain activity of the host (Lee and Hase, 2014).

The host gut-microbial relationship is dynamic and highly susceptible to numerous environmental factors, especially diet, which determines both the overall taxonomic composition and the metabolic activity of the large bowel ecosystem. Resistant starch (RS) is the fraction of ingested starch that escapes enzymatic digestion by endogenous enzymes in the upper gastrointestinal tract and passes into the cecum and colon, where it can be fermented by cecal and colonic microbes (Topping and Clifton, 2001; Bach Knudsen et al., 2012). The inclusion of raw potato starch (RPS) to the diet of pigs could increase the amount of starch entering the large bowel due to the native granular structure of this starch (Martinez-Puig et al., 2003; Fang et al., 2014). Currently, the potato becomes the fourth staple crops for human food in China, thus it is necessary to understand the role of potato starch in the health of humans.

Although high RS diets were associated with lower apparent total tract digestibility of nutrients (Gerrits et al., 2012) and lower feed efficiency (Regmi et al., 2011), there is a growing interest in including both RS and other sources of non-digestible carbohydrates into pig diets for the potential prebiotic properties (Bach Knudsen et al., 2012). RS can be fermented by the gut microflora, providing a source of carbon and energy for bacteria present in this anaerobic environment (Macfarlane and Englyst, 1986) and thus potentially altering the composition of the microflora and its metabolic activities. RS is known to be fermented to a large extent by microbiota in the colon, resulting in the production of short-chain fatty acids (SCFAs), which provides an important link between dietary RS consumption and health benefits (Roy et al., 2006; Hamer et al., 2008; Sekirov et al., 2010). However, so far, information on the effects of RS on other microbial metabolites in the large intestine of pigs is limited.

Because of its similar homology to human, the pig has been regarded as an ideal model for the study of human nutrition (Guilloteau et al., 2010), however, compared with the human, the pig has a quite larger cecum where a part of starches can be fermented by microbiota before entering the colon. Thus, it is supposed that the modulation of microbiota and their metabolism by RS may be different between cecum and colon. Therefore, by feeding the RPS diet to pigs in a long-term, the purpose of this study was to investigate the effects of RS on microbial composition and microbial metabolites both in the cecum and colon, and to reveal the correlation between microbes and metabolites.

## Materials and methods

### Ethics statement

The experiment was approved and conducted under the supervision of the Animal Care and Use Committee of Nanjing Agricultural University (Nanjing, Jiangsu province, China). All pigs were raised and maintained on a local commercial farm under the care of the Animal Care and Use Guidelines of Nanjing Agricultural University.

### Animals, housing, diets, and sampling

Thirty-six 70-day Duroc × Landrace × Large White growing barrows were randomly allocated into corn starch (CS) and RPS diet groups. Each group consisted of six pens (replicates), with three pigs per pen. Pigs in the CS group were fed a corn/soybean-based diet, which reached the nutrient requirement of the NRC (1998) (Table 1). In the RPS group, 230 and 280 g/kg of purified CS was replaced with purified RPS for growing (70 days) and finishing (120 days) pigs, respectively. The pigs had free access to feed and water during the 100-d animal trial.

**Table 1:** **Composition and nutrient analysis of experimental diets (as-fed basis)**.

**Diets**	**Growing pigs**		**Finishing pigs**	
	**CS**	**RPS**	**CS**	**RPS**
**INGREDIENTS (g/kg)**				
Corn starch	230.0	–	280.0	–
Raw potato starch	–	230.0	–	280.0
Corn	360.0	360.0	360.0	360.0
Wheat bran	90.0	90.0	120.0	120.0
Soybean meal	250.0	250.0	210.0	210.0
Extruded soybean	30.0	30.0	–	–
Soybean oil	8.00	8.00	–	–
Dicalcium phosphate	9.80	9.80	8.80	8.80
Limestone	7.80	7.80	7.70	7.70
Salt	3.00	3.00	3.00	3.00
Vitamin and mineral premix^a^	10.0	10.0	10.0	10.0
L-Lysine	1.00	1.00	0.50	0.50
L-Methionine	0.40	0.40	–	–
**NUTRIENT ANALYSIS (g/kg)**				
CP	174.5	174.5	147.3	147.3
Starch	505.6	504.5	550.2	549.5
Resistant starch	6.40	133.5	5.20	153.5
Ash	72.1	73.2	61.0	61.6
NDF	95.77	95.78	102.5	102.6

a *This mineral and vitamin premix (1%) supplies per kg diet as follows: VA 11 000 IU, VD3 1 000 IU, VE 16 IU, VK1 1mg, VB1 0.6 mg, VB2 0.6 mg, d-pantothenic acid 6 mg, nicotinic acid 10 mg, VB12 0.03 mg, folic acid 0.8 mg, VB6 1.5 mg, choline 800 mg, Fe 165 mg, Zn 165 mg, Cu 16.5 mg, Mn 30 mg, Co 0.15 mg, I 0.25 mg, Se 0.25 mg*.

On day 170 (of age), one pig from each pen was randomly selected and slaughtered when it approached the weight of 105 kg. Before slaughtering, the feed was withheld from the pigs for 12 h. The pigs were slaughtered via electrical stunning followed by exsanguination. The digesta were collected from the cecum and proximal colon, then stored in liquid nitrogen for further microbiome and metabolome analysis.

### DNA extraction and 16S rRNA gene amplicon pyrosequencing

The total genomic DNA was extracted from both cecal and colonic samples using a commercially stool DNA extraction kit according to the instructions of the manufacturer (QIAamp DNA Stool Mini Kit, Qiagen, Hilden, Germany). The DNA concentration was determined using a Nano-Drop 1000 spectrophotometer (Thermo Scientific Inc., Wilmington, DE, USA). Bacterial universal primers (8F/533R) were used for the amplification of the V1–V3 region of the bacterial 16S rRNA gene and subsequent pyrosequencing of the PCR products (Baker et al., 2003). The PCR was performed according to the description of a previous study (Bian et al., 2013). All PCR products were purified with a commercial DNA gel extraction kit (Axygen, China). PCR amplicons were sequenced by the 454 GS FLX Titanium chemistry at the Majorbio Bio-Pharm Technology (Shanghai, China).

### Sequence analysis

Sequences underwent standard quality control and were split into libraries using the default parameters in Quantitative Insights Into Microbial Ecology. The operational taxonomic units (OTUs) were clustered with a 97% similarity cutoff using UPARSE (version 7.1; http://drive5.com/uparse/), and the chimeric sequences were identified and removed using UCHIME. The phylogenetic affiliation of each 16S rRNA gene sequence was analyzed using the RDP Classifier (http://rdp.cme.msu.edu/) against the Silva (SSU115) 16S rRNA database, with a confidence threshold of 70%. The richness estimators [abundance-based coverage estimator (ACE) and the bias-corrected Chao], and the diversity indices (Shannon and Simpson diversity) were calculated using the MOTHUR program (http://www.mothur.org). The raw pyrosequencing reads were submitted to Sequencing Read Archive (SRA) database under the accession id: SRP067915.

### Sample preparation for GC-MS analysis

The GC-MS analysis was conducted according to the description of a previous study (Mao et al., 2016). Briefly, the cecal and colonic contents (approximately 1.0 g) were mixed with 3 ml of H_2_O at 4°C. After centrifugation at 10,000 *g* for 10 min, 50 μl of the supernatant was added with 200 μl of methanol containing 12.5 μg/ml of [^13^C_2_]-myristic acid, maintained at 4°C for 1 h, and centrifuged at 20,000 *g* for 10 min. The supernatant (100 μl) was evaporated to dryness in a SpeedVac concentrator (Savant Instruments, Framingdale, NY, USA). The dried analytes were methoximated and trimethylsilylated as described by Lu et al. (2008). After trimethylsilylation for 1 h at room temperature, 30 μl of n-heptane were added into each GC vial with methyl stearate (10 μg/ml) for quality control. The final mixture was vigorously vortexed for 1 min and was then ready for GC-MS analysis.

### Gas chromatograph-mass spectrometry (GC-MS) analysis of metabolite profiles

The derivatized sample (1.0 μl) was immediately injected by an autosampler into an Agilent 6890 GC system coupled with a fused-silica capillary column (10 m × 0.18 mm i.d.) and chemically bonded with 0.18 μm DB-5 stationary phase (J&W Scientific, Folsom, CA, USA). Helium was used as the carrier gas at a constant flow rate of 1.0 ml/ min through the column. The column temperature was initially held at 70°C for 2 min, then ramped to 310°C at a rate of 30°C/min, and finally held for 2 min. The transfer line temperature and ion source temperature were 250°C and 200°C, respectively. Mass fragmentation was generated with an electron beam at 70 eV. Mass data were collected in a full-sacn mode (m/z 50 to 800). Following a solvent delay of 170 s, the detector voltage was set to -1650 V.

### GC-MS data acquisition and processing

After the raw data was collected, identification of the compounds were achieved by comparison of the mass spectrum and retention indices of all the detected compounds with their reference standards and database in the National Institute of Standards and Technology Library 2.0 (2008) and NEW Wiley 9 mass spectra library database. The relative quantitative peak areas of each detected peak were normalized to [^13^C2]-myristic acid, the stable isotope IS, and the data were arranged on a two-dimensional matrix consisting of arbitrary sample names (observations) and peak area (variables). Multivariate statistical analysis was conducted with SIMCA-P+ version 13.0 software package (Umetrics, Umea, Sweden). The acquired GC/MS data were processed with partial least squares projection to latent structures and discriminant analysis (PLS–DA). The metabolites with variable importance projection (VIP) values of 1.0 and *P*-values of 0.05 (threshold) were considered as metabolites that could discriminate between two dietary groups. The impact of RPS on metabolic pathways and metabolite set enrichment analysis was evaluated based on an online tool (http://www.metaboanalyst.ca/MetaboAnalyst/faces/ModuleView.xhtml) (Xia et al., 2009).

### Statistical analysis

Data were analyzed by SPSS 17.0 as a randomized block design, considering the diet as the main effect and the replicate as a block. The effects of diet on microbial metabolites in the cecum and colon of pigs were tested for significance using Student's *t*-test. The effects of diet and gut segment on the bacterial abundance were tested for significance using a two-way ANOVA program. *P*-values were corrected for multiple testing by using a false-discovery rate (*Q*-value) method (Benjamini and Hochberg, 1995). Significant differences were declared when *P* < 0.05. Correlations between hindgut compounds and bacterial compositions were assessed by Pearson's correlation test using GRAPHPAD PRISM version 5.00 (GRAPHPAD Software, San Diego, CA, USA).

## Results

### Effects of RPS on microbial composition

**Figure 1:**
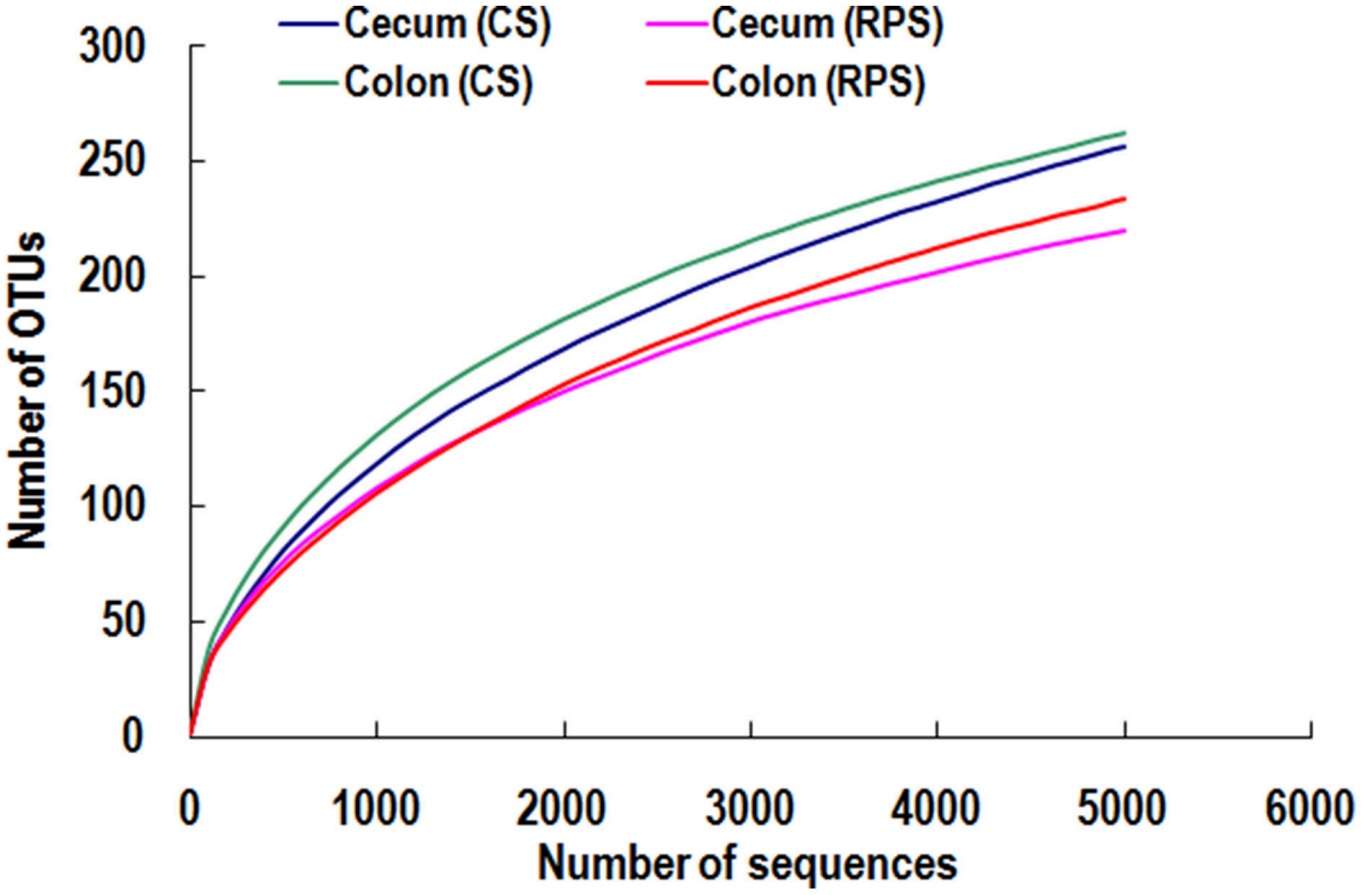
**Rarefaction curves comparing the number of sequences with the number of OTUs found in the 16S rRNA gene libraries from microbiota in the cecum and colon of pigs in the corn starch (CS) and raw potato starch (RPS) groups**.

High-throughput sequencing was performed to compare the microbial communities in the cecum and colon between the two dietary groups. The rarefaction curves generated by MOTHUR plotting the number of sequences by the number of OTUs tended to approach the saturation plateau (Figure 1). The statistical estimates of species richness for 5000-sequence subsets from each sample at a genetic distance of 3% showed that there were no differences in diversity indices (Shannon and Simpson) and richness estimators (ACE and Chao) of the cecal microbiota between the two dietary groups. However, the richness estimators (Chao) in the colonic microbiota significantly decreased by the RPS dietary treatment (Table 2).

**Table 2:** **Diversity estimation of the 16S rRNA gene libraries from microbiota in the cecum and colon of pigs fed corn starch (CS) and raw potato starch (RPS) diets**.

**Group**		**Ace**	**Chao**	**Shannon**	**Simpson**
Cecum	CS	403.17 ± 98.33	368.93 ± 59.68	3.390 ± 0.496	0.079 ± 0.034
	RPS	294.18 ± 52.79	283.13 ± 56.76	3.241 ± 0.486	0.125 ± 0.076
	*P*-value	0.257	0.460	0.787	0.691
Colon	CS	399.08 ± 98.16	355.58 ± 107.83	3.474 ± 0.656	0.098 ± 0.071
	RPS	342.46 ± 33.20	316.03 ± 37.12	3.571 ± 0.428	0.082 ± 0.043
	*P*-value	0.061	0.048	0.646	0.254

**Figure 2:**
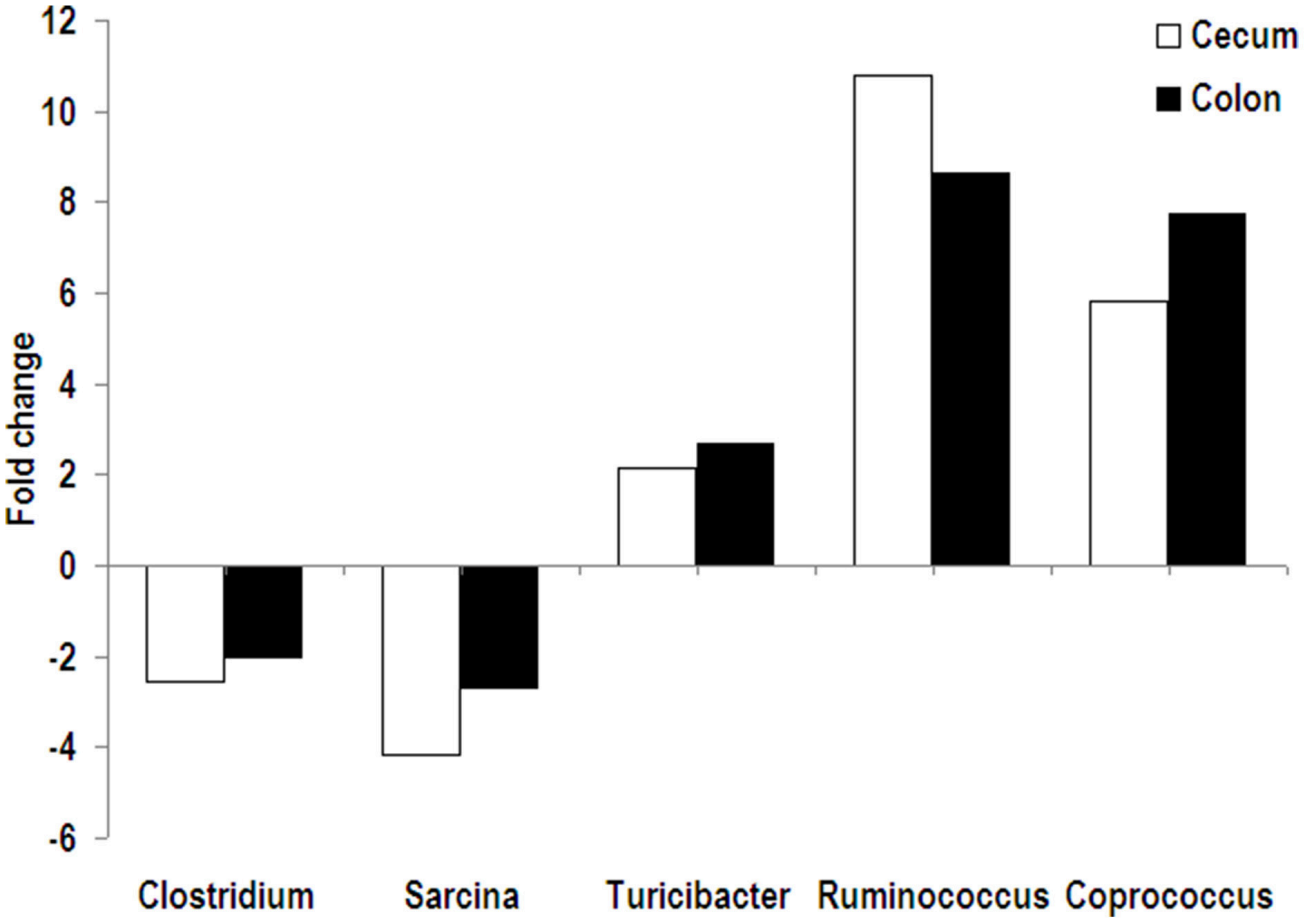
**Fold change of relative abundance (RPS vs. CS)**. Bacterial genera were significantly affected by dietary RPS in the cecum and colon of pigs.

Firmicutes was predominant in both cecal and colonic microbiota of pigs with an abundance of higher than 96%, followed by the phyla Proteobacteria and Spirochaeta. No significant difference in relative abundance at the phylum level was found between the RPS and CS groups. At the genus level, *Streptococcus*, uncultured Ruminococcaceae, and *Lactobacillus* were the predominant genera in the cecal and colonic microbiota of pigs in two dietary groups. In the cecum, compared with the CS group, the relative abundance of *Clostridium* and *Sarcina* was significantly decreased in the RPS group, while there was a trend of increased abundance of *Coprococcus* in pigs fed RPS (Figure 2). In the colon, the relative abundance of *Turicibacter* in pigs fed the RPS diet was significantly higher than that in pigs fed the CS diet. Compared with the CS group, *Ruminococcus* and *Coprococcus* tended to increase in abundance, while *Sarcina* and *Dorea* were significantly declined in the RPS group. At the OTU level, consumption of the RPS diet significantly decreased Lactobacillus-, Clostridium-, and Sarcina-related OTUs, and increased Blautia-, Ruminococcaceae-, Ruminococcus-, Subdoligranulum-, and Turicibacter-related OTUs in the cecum and colon of pigs as compared with the CS diet (Table 3).

**Table 3:** **Relative abundances of OTUs (>0.05%) in the cecal and colonic contents of pigs that were significantly affected by gut segment or diet**.

**OTU ID**	**Cecum**		**Colon**			***P-*****value**		***Q*****-value**		**Phylogenetic group^b^**
	**CS**	**RPS**	**CS**	**RPS**	**SEM^a^**	**Gut segment**	**Diet**	**Gut segment**	**Diet**	
OTU329	0.152	0.392	0.145	0.469	0.041	0.673	0.002	0.935	0.011	p:Firmicutes
OTU959	5.678	0.712	3.171	0.472	0.839	0.423	0.034	0.879	0.050	s:*Lactobacillus prophage* Lj928
OTU814	4.390	0.773	2.639	0.425	0.649	0.428	0.036	0.879	0.050	s:*Lactobacillus prophage* Lj929
OTU643	1.602	0.293	1.208	0.223	0.225	0.613	0.019	0.935	0.043	s:*Lactobacillus prophage* Lj930
OTU363	0.031	0.086	0.043	0.085	0.010	0.800	0.030	0.935	0.050	o:Bacillales
OTU745	0.030	0.122	0.018	0.189	0.025	0.601	0.018	0.935	0.043	f:Christensenellaceae
OTU658	0.077	0.000	0.299	0.000	0.033	0.105	0.009	0.585	0.033	f:Christensenellaceae
OTU411	7.161	1.672	8.439	2.849	0.542	0.271	< 0.001	0.879	0.004	g:*Sarcina*
OTU24	2.396	0.636	2.048	1.068	0.194	0.915	0.002	0.935	0.011	g:*Sarcina*
OTU4	0.458	0.095	0.566	0.187	0.041	0.239	< 0.001	0.847	0.004	g:*Sarcina*
OTU650	7.474	1.872	6.918	2.678	0.617	0.920	0.001	0.935	0.008	g:*Clostridium*
OTU230	2.508	0.707	1.871	0.763	0.221	0.518	0.004	0.935	0.017	g:*Clostridium*
OTU272	0.000	0.270	0.000	0.291	0.061	0.932	0.032	0.935	0.050	g:*Blautia*
OTU30	0.781	0.211	0.659	0.299	0.060	0.890	0.001	0.935	0.008	f:Lachnospiraceae
OTU852	0.003	0.200	0.005	0.353	0.058	0.511	0.029	0.935	0.050	f:Lachnospiraceae
OTU934	0.018	0.064	0.030	0.170	0.021	0.177	0.039	0.847	0.050	f:Lachnospiraceae
OTU911	0.118	0.000	0.201	0.003	0.035	0.552	0.037	0.935	0.050	f:Lachnospiraceae
OTU380	0.003	0.132	0.025	0.144	0.029	0.770	0.046	0.935	0.054	g:*Pseudobutyrivibrio*
OTU499	1.152	0.825	3.126	1.938	0.220	0.002	0.100	0.078	0.108	f:Peptostreptococcaceae
OTU683	0.804	0.460	1.194	1.253	0.105	0.011	0.505	0.143	0.532	f:Peptostreptococcaceae
OTU607	0.261	0.414	0.460	0.605	0.032	0.007	0.032	0.137	0.049	f:Peptostreptococcaceae
OTU369	0.214	0.432	0.154	0.394	0.040	0.548	0.01	0.935	0.033	f:Peptostreptococcaceae
OTU210	0.030	0.041	0.094	0.077	0.011	0.034	0.896	0.228	0.896	f:Peptostreptococcaceae
OTU324	0.049	1.474	0.051	1.582	0.333	0.935	0.038	0.935	0.050	f:Ruminococcaceae
OTU860	0.817	0.121	0.698	0.087	0.117	0.747	0.011	0.935	0.033	f:Ruminococcaceae
OTU636	0.237	0.018	0.159	0.035	0.031	0.629	0.012	0.935	0.033	f:Ruminococcaceae
OTU175	0.108	0.028	0.051	0.016	0.014	0.220	0.049	0.847	0.055	f:Ruminococcaceae
OTU872	0.000	0.236	0.011	0.441	0.063	0.402	0.015	0.879	0.039	g:*Ruminococcus*
OTU703	0.000	0.137	0.000	0.322	0.046	0.322	0.02	0.879	0.043	g:*Ruminococcus*
OTU274	0.008	0.053	0.020	0.135	0.019	0.228	0.046	0.847	0.054	g:*Ruminococcus*
OTU527	0.000	0.130	0.000	0.302	0.048	0.384	0.036	0.879	0.050	g:*Subdoligranulum*
OTU195	0.000	0.086	0.000	0.196	0.033	0.419	0.047	0.879	0.054	g:*Subdoligranulum*
OTU364	3.614	8.030	3.130	8.022	0.967	0.900	0.026	0.935	0.050	g:*Turicibacter*
OTU615	1.140	2.179	0.738	2.277	0.273	0.784	0.029	0.935	0.050	g:*Turicibacter*
OTU477	0.017	0.073	0.024	0.099	0.009	0.372	0.001	0.879	0.008	g:*Turicibacter*
OTU159	0.416	0.088	0.356	0.131	0.041	0.920	0.003	0.935	0.015	o:Selenomonadales
OTU340	0.340	0.221	0.004	0.000	0.061	0.033	0.619	0.228	0.635	g:*Pseudomonas*
OTU970	0.106	0.000	0.152	0.000	0.029	0.695	0.040	0.935	0.050	g:*Leeia*
OTU480	0.353	0.586	0.505	1.071	0.070	0.035	0.010	0.228	0.033	d:Bacteria

a *SEM, standard error of means, n = 6*.

b *The consensus sequence of each OTU was annotated to the closest lineage using MOTHUR program against the SILVA 16S rRNA reference database. s, species; g, genus; f, family; o, order, p, phylum*.

### Effects of RPS on metabolite profiles

A typical total-ion-current chromatogram showed several hundred peaks in a single analysis. After deconvolution of the chromatograms, the quantitative and qualitative information was obtained. In general, a total of 180 non-targeted peaks/metabolites were detected. In a comparison with authentic reference standards or reference compounds in available libraries, 92 compounds were annotated by GC-MS. These metabolites, including organic acids, amino acids, fatty acids, saccharides, amines, and lipids, are involved in multiple biochemical processes in the hindgut of pigs.

**Figure 3:**
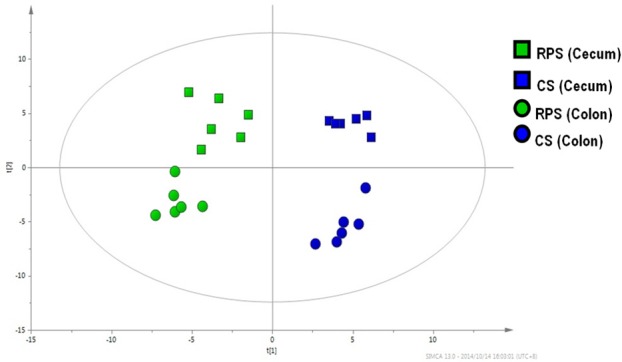
**Partial least squares discriminant analysis (PLS-DA)**. PLS-DA of microbial metabolites in cecal and colonic contents from pigs fed corn starch (CS) and raw potato starch (RPS).

**Figure 4:**
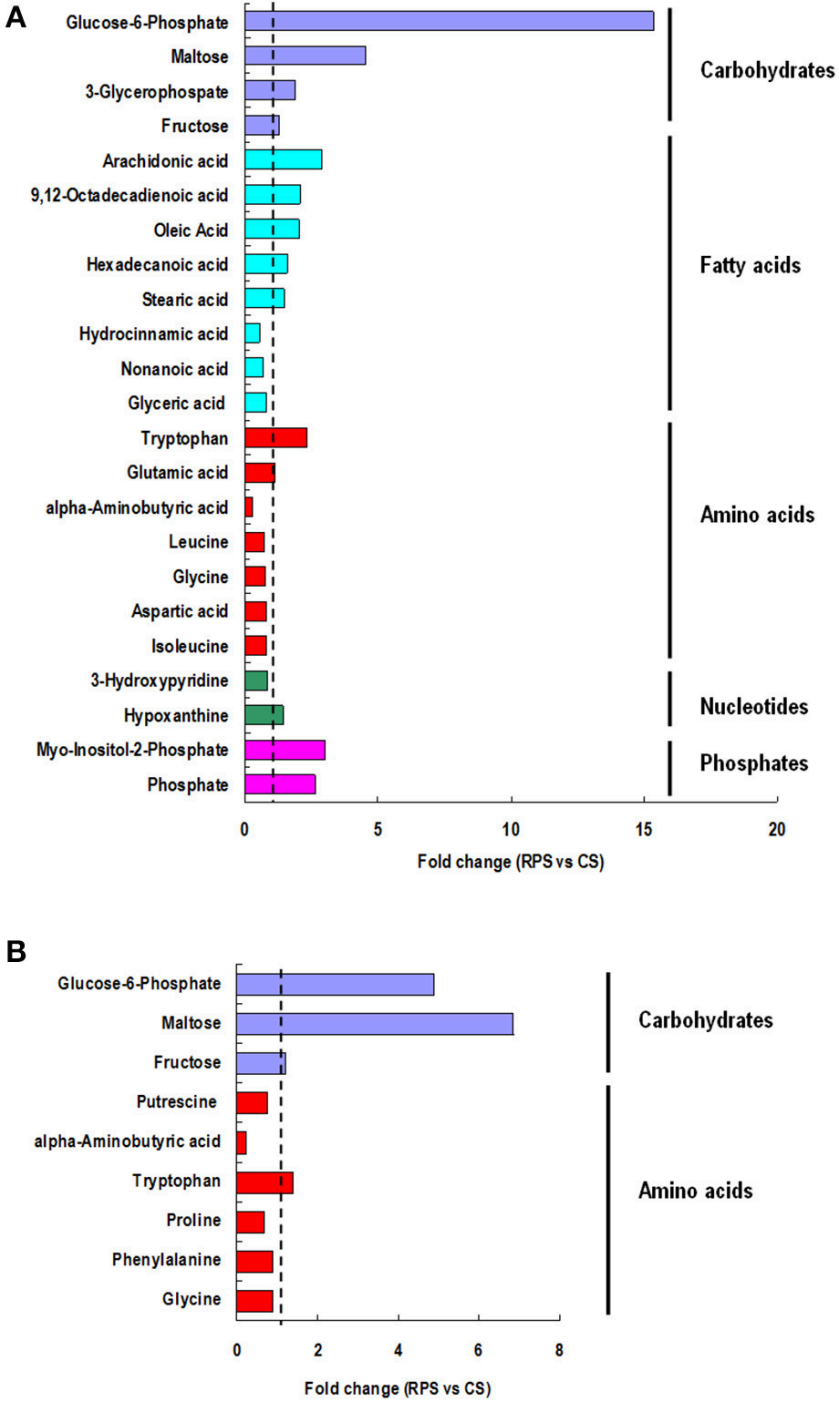
**Significant compounds**. Metabolites accountable for class discrimination with VIP > 1, fold change > 1.5, and *P* < 0.05 were listed. **(A)** cecum; **(B)** colon.

**Figure 5:**
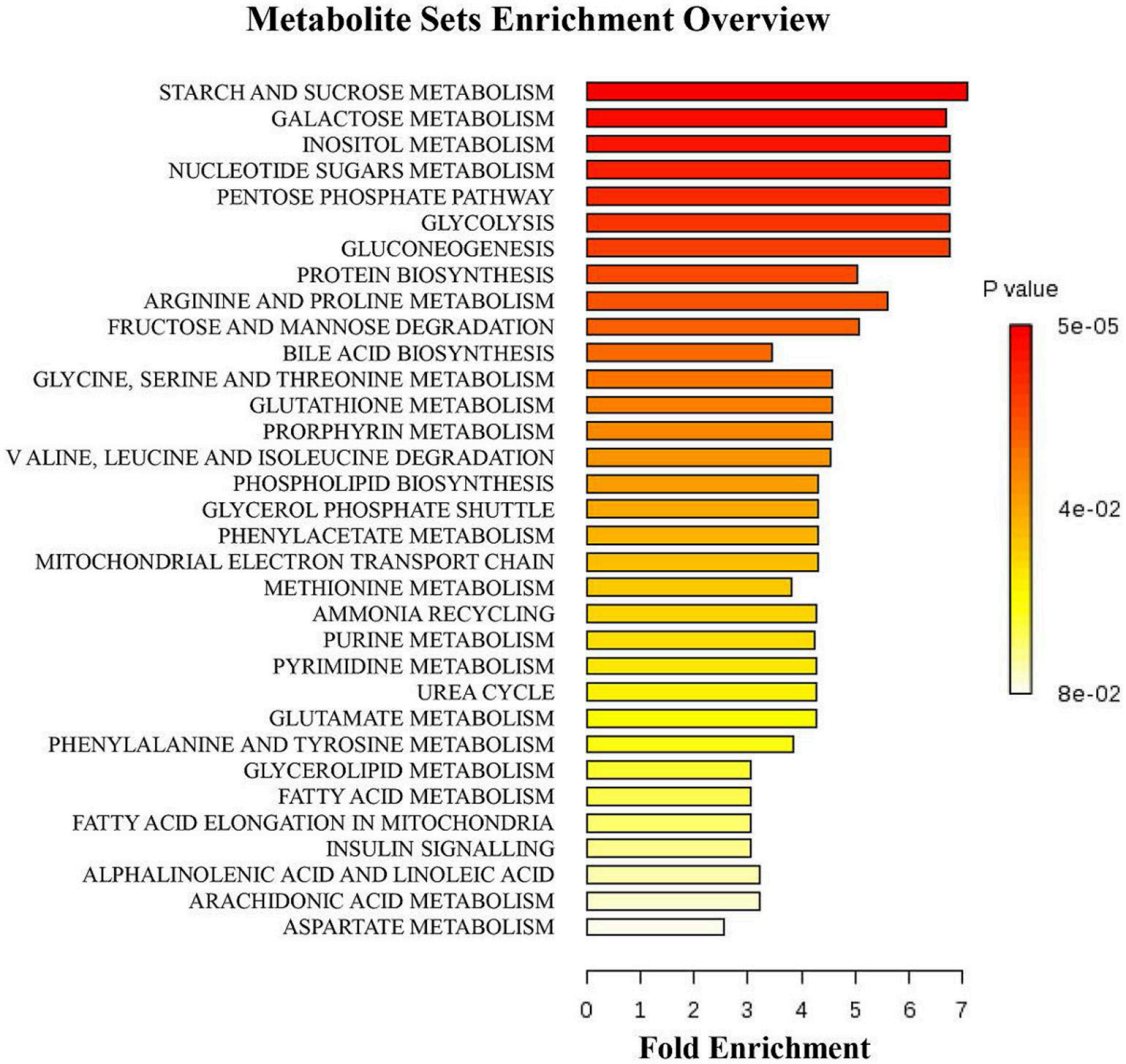
**Metabolic pathway enrichment analysis**. Overview of metabolites that were enriched in pigs fed the RPS diet compared to the CS diet.

A multivariate analysis method, the PLS-DA, was used to identify the key compounds responsible for the differentiation. The variation in the microbial metabolites could be differentiated readily according to different starch sources in the diet, with an excellent separation of microbial metabolites by cecum and colon (Figure 3). To identify which compounds were responsible for this difference between the two dietary groups, the parameters of VIP > 1, fold change > 1.5, and *P* < 0.05 were used as criteria. In the cecum, 14 compounds (glucose-6-phosphate, maltose, 3-glycerophosphate, fructose, arachidonic acid, 9,12-octadecadienoic acid, oleic acid, hexadecanoic acid, stearic acid, tryptophan, glutamic acid, hypoxanthine, myo-inositol-2-phosphate, and phosphate) were enriched, while nine (hydrocinnamic acid, nonanoic acid, glyceric acid, alpha-aminobutyric acid, leucine, glycine, aspartic acid, isoleucine and 3-hydroxypyridine) were reduced in the RPS group compared with the CS group (Figure 4A). In the colon, four of these compounds (maltose, glucose-6-phosphate, fructose, and tryptophan) were enriched, whereas five (alpha-aminobutyric acid, proline, putrescine, phenylalanine, and glycine) were reduced in pigs fed the RPS diet compared with the CS diet (Figure 4B). Further metabolic pathway enrichment analysis showed that the high RS diet had significant effects on starch and sucrose metabolism, protein synthesis, lipid metabolism, sugar fermentation solution, the pentose phosphate pathway, inositol phosphate metabolism, and nucleotide metabolism (Figure 5).

### Correlation between microbial community and their metabolites

**Figure 6:**
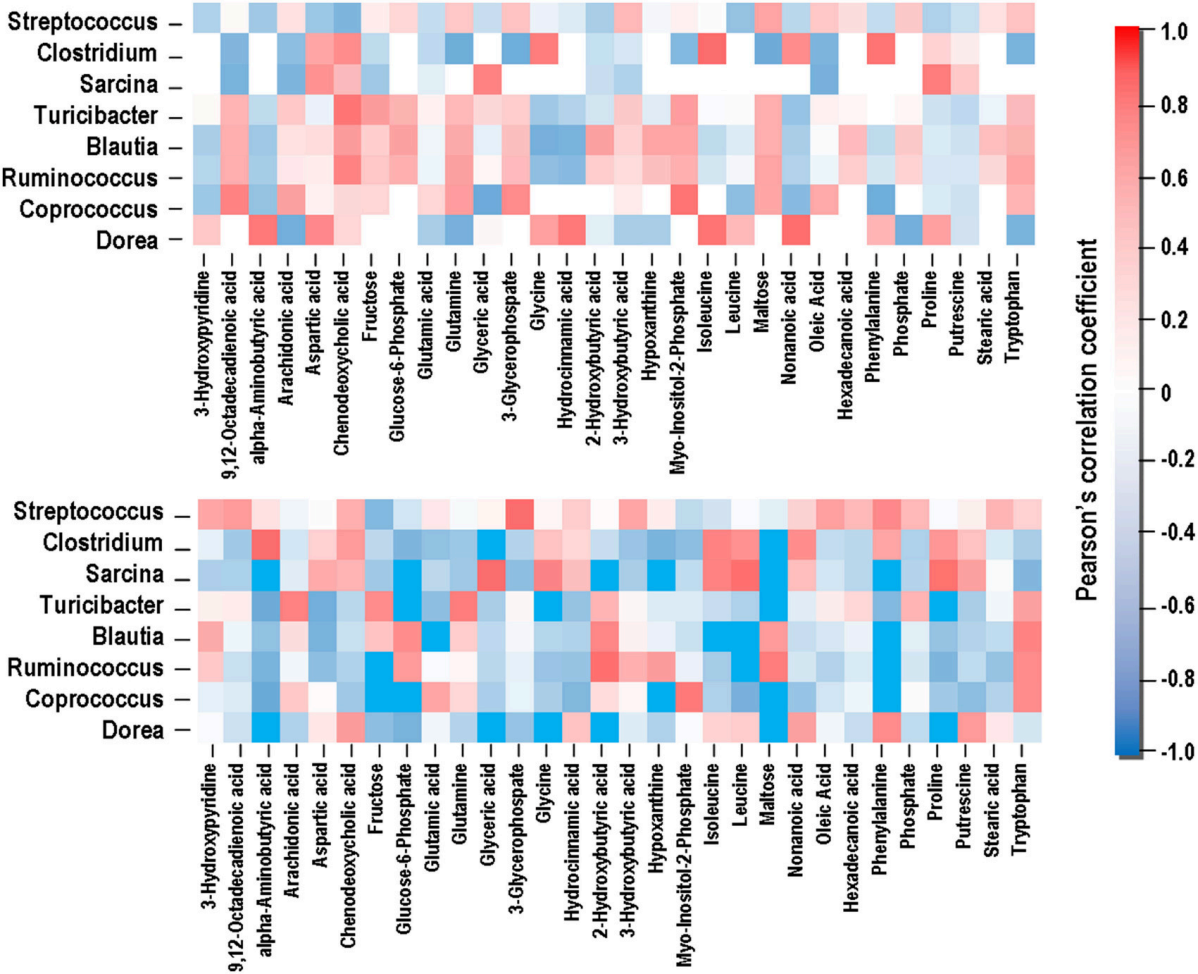
**Correlation between the gut microbiome and metabolites**. The color is according to the Pearson correlation coefficient distribution; red represents significant positive correlation (*P* < 0.05), blue represents significantly negative correlation (*P* < 0.05), and white represents that the correlation was not significant (*P* > 0.05).

Compounds with VIP > 1 and bacterial genera significantly affected by dietary treatment were used for the Pearson's correlation analysis. As shown in Figure 6, the relative abundance of genera *Coprococcus, Ruminococcus*, and *Turicibacter* was positively correlated with maltose, 6-phosphate glucose, glutamate, and glutamine (*P* < 0.05), and negatively correlated with putrescine and 3-hydroxypyridine (*P* < 0.05). Genera *Clostridium* and *Sarcina* showed negative correlations with maltose, 6-phosphate glucose, glutamate, and glutamine (*P* < 0.05), and positive correlations with putrescine and 3-hydroxypyridine (*P* < 0.05).

## Discussion

RPS is gelatinized poorly and hydrolyzed slowly by α-amylase. On this basis, we chose RPS to generate a supply of RS to the large bowels of growing-finishing pigs in the present study. We examined the effects of the long-term intake of a diet high in RS on the microbial composition and metabolomic profiles of the cecum and colon of pigs. Our results revealed that RS modulated the microbial composition in the cecum and colon of pigs, and discriminatively changed the metabolomic profiles in their hindgut.

In the present study, consumption of an RPS diet did not affect the abundance of phyla in the cecum and colon, which is consistent with a previous study in which type 2 RS had no effects on the fecal microbial community at the phylum level (Martínez et al., 2010; Sun et al., 2015). Metzler-Zebeli et al. (2015) found that the diversity, species richness, and phyla abundance in the cecal microbiota of pig were not impacted by the enzymatically modified starch (type 4 RS). However, Sybille et al. found that the cecal microbiota of mice fed HAM-RS2 had a higher proportion of phyla Bacteroidetes, Actinobacteria, and Verrucomicrobia (Sybille et al., 2013). The conflicting results of different studies may be due to the different sources of RS being used in different animal models.

Similar to our previous study by Miseq sequencing (Sun et al., 2015), at the genus level, RS decreased the relative abundances of *Clostridium*-, *Sarcina*-, *Lactobacillus*-, and *Leeia*-related bacteria, and increased the abundances of *Ruminococcus*-, *Subdoligranulum*-, *Solobacterium*-, *Coprococcus*-, and *Turicibacter*-related bacteria in the cecum and colon of pig. *Clostridium* contains common free-living bacteria, as well as important pathogens (Wells and Wilkins, 1996), and *Sarcina* are fastidious Gram-positive anaerobic bacteria that occur in cubical packets of eight or more cells. Edwards et al. (2008) found that *Sarcina* was associated with mucosal hyperemia and hemorrhage in young lambs and calves, while Crowther (1971) found that diet could influence the colonization of *Sarcina* in the human intestine. The consequent decreased abundance of *Clostridium* and *Sarcina* may benefit the health of the intestine.

The genus *Ruminococcus*, which belongs to *Clostridium* cluster IV, was significantly higher in the hindgut following the RPS diet. Some studies indicated this genus plays an important part in the degradation of RS (Flint et al., 2008; Chassard et al., 2012). Due to its central role in the fermentation of RS, Ze et al. (2012) concluded that Ruminococcus bromii is a keystone species for the degradation of RS in the human colon. Recent studies have reported that R. bromii was largely increased in the large intestine of pigs and humans consuming an RS diet (Abell et al., 2008; Walker et al., 2011; Umu et al., 2015). The abundance of genus *Coprococcus* was also significantly higher in the hindgut with the RPS diet. Louis and Flint (2009) showed that strains related to *Coprococcus* were listed in major butyrate-producing bacteria isolated from the human colon. As is well known, *Clostridium* clusters XIVa and IV are regarded as two of the most predominant populations in the pig fecal microbiota and contain numerous butyrate-producing bacteria (Pryde et al., 2002; Louis et al., 2007). The increase in the abundance of butyrate-producing bacteria is in agreement with the fact that an RPS diet significantly increased the concentrations of butyrate and total SCFA, as was shown in our preliminary study (Fang et al., 2014).

Microbial fermentation with different substrates leads to different microbial metabolic processes and metabolite profiles (Louis et al., 2007). Martinez-Puig et al. (2003) found that the digestibility of starch and protein in the ileum was significantly lower in RPS-fed pigs, but increased from ileum to rectum, and most starch was extensively fermented in the cecum and proximal colon. Similarly, our previous study also found that the starch content in the ileum, cecum, and colon of pigs fed the RPS diet was significantly higher than that in pigs fed the CS diet (Fang et al., 2014). Thus, it is not strange to observe the enriched glucose-6-phosphate and maltose in the RPS group compared with the CS group. This is also consistent with the increase of the abundance of *Ruminococcus*, the main starch degradator in the cecum and colon of pigs. As a result, the starch was fermented into SCFAs, which is in agreement with the fact that the RPS diet significantly increased the concentrations of butyrate and total SCFAs in our preliminary study (Fang et al., 2014).

Meanwhile, the concentrations of five unsaturated fatty acids in the RPS group, including arachidonic acid, 9,12-octadecadienoic acid, oleic acid, hexadecanoic acid, and stearic acid, were significantly higher than in the CS group, possibly indicating a lower absorption of unsaturated fatty acids in the cecum. RS has recently been demonstrated to regulate lipid metabolism, and decreasing the absorption of fatty acids may be one of its approaches (Lee et al., 2012). In addition, the concentration of some amino acid relatives was significantly decreased in the RPS group, showing fewer nitrogen sources existed for fermentation. The possible reason is that the decrease in the digestibility of starch in the ileum results in more carbon source, and increases the carbon:nitrogen ratio of substrates for microbes in the cecum and colon. Furthermore, the production of a potentially harmful by-product (putrescine) of protein fermentation was also decreased, which is consistent with previous research conducted in humans and indicates that RS has a favorable effect on the luminal environment (Birkett et al., 1996). With the improvement of the living standard, the human gut health issues such asenteritis, colon and rectum cancer attach our attention. By using the animal models, more and more studies showed that dietary RS plays benefit roles in the gut health of humans by mainly modulating the microbiota and their meatbolites, which can provide guidance to people's diet.

PLS-DA showed that the metabolite profiles were different between the cecum and colon, and the amount of discriminating compounds in the cecum was higher than in the colon. The possible reason is that the un-degraded starch in the small intestine first enters the cecum and is extensively fermented by microbiota and as a result, the residual starch is much lower in the colon. The differences in the microbiota and their metabolites between the cecum and colon should be considered when using a pig model for human studies.

In conclusion, the present study investigated the microbiome-metabolome responses in the cecum and colon of pigs fed a high RS diet. We found that long-term consumption of the RPS diet discriminatively altered the microbial composition and modulated the metabolic pathway of microbial metabolism in the cecum and colon of pig. These alterations may help us to understand the beneficial impacts of long-term intake of PRS on the nutrition and health of animals and humans.

## Author contributions

Conceived and designed the experiments: YS, WZ. Performed the experiments: YS, YS. Analyzed the data: YS, YS. Wrote the paper: YS, YS.

### Conflict of interest statement

The authors declare that the research was conducted in the absence of any commercial or financial relationships that could be construed as a potential conflict of interest.

## References

[B1] Abell G. Cooke C. Bennett C. Conlon M. McOrist A. L. (2008). Phylotypes related to ruminococcus bromii are abundant in the large bowel of humans and increase in response to a diet high in resistant starch. FEMS Microbiol. Ecol. 66, 505–515. 10.1111/j.1574-6941.2008.00527.x 18616586

[B2] Bach Knudsen K. Hedemann M. Lærke H. (2012). The role of carbohydrates in intestinal health of pigs. Anim. Feed Sci. Tech. 173, 41–53. 10.1016/j.anifeedsci.2011.12.020

[B3] Baker G. Smith J. Cowan D. (2003). Review and re-analysis of domain-specific 16S primers. J. Microbio. Meth. 55, 541–555. 10.1016/j.mimet.2003.08.009 14607398

[B4] Benjamini Y. Hochberg Y. (1995). Controlling the false discovery rate: a practical and powerful approach to multiple testing. J. R. Stat. Soc. B. 57, 289–300.

[B5] Bian G. Ma L. Su Y. Zhu W. (2013). The microbial community in the feces of the white rhinoceros (*Ceratotherium simum*) as determined by barcoded pyrosequencing analysis. PLoS ONE 8:e70103. 10.1371/journal.pone.0070103 23922920 PMC3724812

[B6] Birkett A. Muir J. Phillips J. Jones G. O'Dea K. (1996). Resistant starch lowers fecal concentrations of ammonia and phenols in humans. Am. J. Clin. Nutr. 63, 766–772. 8615362 10.1093/ajcn/63.5.766

[B7] Chassard C. Delmas E. Robert C. Lawson P. Bernalier-Donadille A. (2012). *Ruminococcus champanellensis* sp. nov., a cellulose-degrading bacterium from human gut microbiota. Int. J. Syst. Evol. Microbiol. 62, 138–143. 10.1099/ijs.0.027375-0 21357460

[B8] Crowther J. (1971). *Sarcina ventriculi* in human faeces. J. Med. Microbiol. 4, 343–350. 10.1099/00222615-4-3-343 5116255

[B9] Edwards G. Woodger N. Barlow A. Bell S. Harwood D. Otter A.. (2008). Sarcina-like bacteria associated with bloat in young lambs and calves. Vet. Rec. 163, 391–393. 10.1136/vr.163.13.391 18820327

[B10] Fang L. Jiang X. Su Y. Zhu W. (2014). Long-term intake of raw potato starch decreases back fat thickness and dressing percentage but has no effect on the longissimus muscle quality of growing–finishing pigs. Livest. Sci. 170, 116–123. 10.1016/j.livsci.2014.10.004

[B11] Flint H. J. Bayer E. Rincon M. Lamed R. White B. A. (2008). Polysaccharide utilization by gut bacteria: potential for new insights from genomic analysis. Nat. Rev. Microbiol. 6, 121–131. 10.1038/nrmicro1817 18180751

[B12] Gerrits W. J. Bosch M. W. van den Borne J. J. (2012). Quantifying resistant starch using novel, *in vivo* methodology and the energetic utilization of fermented starch in pigs. J. Nutr. 142, 238–244. 10.3945/jn.111.147496 22223577

[B13] Guilloteau P. Zabielski R. Hammon H. M. Metges C. C. (2010). Nutritional programming of gastrointestinal tract development. Is the pig a good model for man? Nutr. Res. Rev. 23, 4–22. 10.1017/S0954422410000077 20500926

[B14] Hamer H. M. Jonkers D. Venema K. Vanhoutvin S. Troost F. Brummer R. (2008). Review article: the role of butyrate on colonic function. Aliment. Pharmacol. Ther. 27, 104–119. 10.1111/j.1365-2036.2007.03562.x 17973645

[B15] Lee K. Yoo S. Lee H. (2012). The effect of chemically−modified resistant starch, RS type−4, on body weight and blood lipid profiles of high fat diet−induced obese mice. Starch−Stärke 64, 78–85. 10.1002/star.201100057

[B16] Lee W. Hase K. (2014). Gut microbiota-generated metabolites in animal health and disease. Nat. Chem. Biol. 10, 416–424. 10.1038/nchembio.1535 24838170

[B17] Li M. Wang B. Zhang M. Rantalainen M. Wang S. (2008). Symbiotic gut microbes modulate human metabolic phenotypes. Proc. Natl. Acad. Sci. U.S.A 105, 2117–2122. 10.1073/pnas.0712038105 18252821 PMC2538887

[B18] Louis P. Flint H. J. (2009). Diversity, metabolism and microbial ecology of butyrate-producing bacteria from the human large intestine. FEMS Microbiol. Lett. 294, 1–8. 10.1111/j.1574-6968.2009.01514.x 19222573

[B19] Louis P. Scott K. Duncan S. Flint H. (2007). Understanding the effects of diet on bacterial metabolism in the large intestine. J. Appl. Microbiol. 102, 1197–1208. 10.1111/j.1365-2672.2007.03322.x 17448155

[B20] Lu Y. A. J. Wang G. Hao H. Huang Q. Yan B.. (2008). Gas chromatography/time-of-flight mass spectrometry based metabonomic approach to differentiating hypertension- and age-related metabolic variation in spontaneously hypertensive rats. Rapid Commun. Mass Spectrom. 22, 2882–2888. 10.1002/rcm.3670 18720470

[B21] Macfarlane G. Englyst H. (1986). Starch utilization by the human large intestinal microflora. J. Appl. Bacteriol. 60, 195–201. 10.1111/j.1365-2672.1986.tb01073.x 2423494

[B22] Mao S. Huo W. Zhu W. (2016). Microbiome–metabolome analysis reveals unhealthy alterations in the composition and metabolism of ruminal microbiota with increasing dietary grain in a goat model. Environ. Microbiol. 18, 525–541. 10.1111/1462-2920.12724 25471302

[B23] Martínez I. Kim J. Duffy P. R. Schlegel V. L. Walter J. (2010). Resistant starches types 2 and 4 have differential effects on the composition of the fecal microbiota in human subjects. PLoS ONE 5:e15046. 10.1371/journal.pone.0015046 21151493 PMC2993935

[B24] Martinez-Puig D. Pérez J. F. Castillo M. Andaluz A. Anguita M. Morales J.. (2003). Consumption of raw potato starch increases colon length and fecal excretion of purine bases in growing pigs. J. Nutr. 133, 134–139. 12514280 10.1093/jn/133.1.134

[B25] Metzler-Zebeli B. U. Schmitz-Esser S. Mann E. Grüll D. Molnar T. Zebeli Q. (2015). Adaptation of the cecal bacterial microbiome of growing pigs in response to resistant starch type 4. Appl. Environ. Microbiol. 81, 8489–8499. 10.1128/aem.02756-15 26431973 PMC4644661

[B26] NRC (1998). Nutrient Requirements of Swine, 10th Rev. Edn. Washington, DC: The National Academies Press.

[B27] Pryde S. Duncan S. Hold G. Stewart C. Flint H. (2002). The microbiology of butyrate formation in the human colon. FEMS Microbiol. Lett. 217, 133–139. 10.1111/j.1574-6968.2002.tb11467.x 12480096

[B28] Rajilić−Stojanović M. Smidt H. De Vos W. M. (2007). Diversity of the human gastrointestinal tract microbiota revisited. Environ. Microbiol. 9, 2125–2136. 10.1111/j.1462-2920.2007.01369.x 17686012

[B29] Regmi P. R. van Kempen T. A. Matte J. J. Zijlstra R. T. (2011). Starch with high amylose and low *in vitro* digestibility increases short-chain fatty acid absorption, reduces peak insulin secretion, and modulates incretin secretion in pigs. J. Nutr. 141, 398–405. 10.3945/jn.110.132449 21248198

[B30] Roy C. C. Kien C. L. Bouthillier L. Levy E. (2006). Short-chain fatty acids: ready for prime time? Nutr. Clin. Pract. 21, 351–366. 10.1177/0115426506021004351 16870803

[B31] Sekirov I. Russell S. L. Antunes L. C. (2010). Finlay BB. Gut microbiota in health and disease. Physiol. Rev. 90, 859–904. 10.1152/physrev.00045.2009 20664075

[B32] Sun Y. Zhou L. Fang L. Su Y. Zhu W. (2015). Responses in colonic microbial community and gene expression of pigs to a long-term high resistant starch diet. Front. Microbiol. 6:877. 10.3389/fmicb.2015.00877 26379652 PMC4548152

[B33] Sybille T. June Z. Michael K. Roy M. (2013). The intestinal microbiota in aged mice is modulated by dietary resistant starch and correlated with improvements in host responses. FEMS Microbiol. Ecol. 83, 299–309. 10.1111/j.1574-6941.2012.01475.x 22909308

[B34] Topping D. L. Clifton P. M. (2001). Short-chain fatty acids and human colonic function: roles of resistant starch and nonstarch polysaccharides. Physiol. Rev. 81, 1031–1064. 11427691 10.1152/physrev.2001.81.3.1031

[B35] Umu O. C. O. Frank J. A. Fangel J. U. Oostindjer M. da Silva C. S. Bolhuis E. J.. (2015). Resistant starch diet induces change in the swine microbiome and a predominance of beneficial bacterial populations. Microbiome 3, 16. 10.1186/s40168-015-0078-5 25905018 PMC4405844

[B36] Walker A. W. Ince J. Duncan S. H. Webster L. M. Holtrop G. Ze X.. (2011). Dominant and diet-responsive groups of bacteria within the human colonic microbiota. ISME J. 5, 220–230. 10.1038/ismej.2010.118 20686513 PMC3105703

[B37] Wells C. Wilkins T. (1996). Clostridia: spore forming anaerobic bacilli, in Baron's Medical Microbiology, 4th Edn., ed Baron S. (Galveston, TX: University of Texas Medical Branch), 562–611. 21413315

[B38] Xia J. Psychogios N. Young N. Wishart D. (2009). MetaboAnalyst: a web server for metabolomic data analysis and interpretation. Nucleic Acids Res. 37, W652–W660. 10.1093/nar/gkp356 19429898 PMC2703878

[B39] Ze X. Duncan S. H. Louis P. Flint H. J. (2012). *Ruminococcus bromii* is a keystone species for the degradation of resistant starch in the human colon. ISME J. 6, 1535–1543. 10.1038/ismej.2012.4 22343308 PMC3400402

[B40] Zoetendal E. Rajiliæ-Stojanoviæ M. De Vos W. (2008). High-throughput diversity and functionality analysis of the gastrointestinal tract microbiota. Gut 57, 1605–1615. 10.1136/gut.2007.133603 18941009

